# Epilepsy in Dravet Syndrome—Current and Future Therapeutic Opportunities

**DOI:** 10.3390/jcm12072532

**Published:** 2023-03-27

**Authors:** Chao Gao, Mikolaj Pielas, Fuyong Jiao, Daoqi Mei, Xiaona Wang, Katarzyna Kotulska, Sergiusz Jozwiak

**Affiliations:** 1 Department of Rehabilitation Medicine, Henan Children’s Hospital, Zhengzhou University, Zhengzhou 450018, China; 2 Department of Neurology and Epileptology, The Children’s Memorial Health Institute, 04-730 Warsaw, Poland; 3 Children’s Hospital, Shaanxi Provincial People’s Hospital, Xi’an 710068, China; 4 Department of Neurology, Henan Children’s Hospital, Zhengzhou University, Zhengzhou 450018, China; 5 Henan Key Laboratory of Children’s Genetics and Metabolic Diseases, Henan Children’s Hospital, Zhengzhou University, Zhengzhou 450018, China; 6 Research Department, The Children’s Memorial Health Institute, 04-730 Warsaw, Poland

**Keywords:** Dravet syndrome, epilepsy, novel therapeutic agents, therapeutic recommendations, efficacy and safety

## Abstract

Dravet Syndrome (DS) is a developmental epileptic encephalopathy characterized by drug-resistant seizures and other clinical features, including intellectual disability and behavioral, sleep, and gait problems. The pathogenesis is strongly connected to voltage-gated sodium channel dysfunction. The current consensus of seizure management in DS consists of a combination of conventional and recently approved drugs such as stiripentol, cannabidiol, and fenfluramine. Despite promising results in randomized clinical trials and extension studies, the prognosis of the developmental outcomes of patients with DS remains unfavorable. The article summarizes recent changes in the therapeutic approach to DS and discusses ongoing clinical research directions. Serotonergic agents under investigation show promising results and may replace less DS-specific medicines. The use of antisense nucleotides and gene therapy is focused not only on symptom relief but primarily addresses the underlying cause of the syndrome. Novel compounds, after expected safe and successful implementation in clinical practice, will open a new era for patients with DS. The main goal of causative treatment is to modify the natural course of the disease and provide the best neurodevelopmental outcome with minimum neurological deficit.

## 1. Introduction and Syndrome Characteristics

Dravet syndrome (DS) is a severe childhood-onset epilepsy syndrome and epileptic encephalopathy (DEE). The first description of the syndrome was made by Charlotte Dravet in 1978 under the name of severe myoclonic epilepsy in infancy; in 1989, the International League Against Epilepsy recognized the disease as a syndrome. The syndrome is considered a rare genetic disease, appearing with an incidence of 1:16,000–1:40,000 [1,2,3,4].

The first symptom of DS is a convulsive seizure appearing in the range of 1 to 18 months, with most of the cases between 4 and 8 months in a previously healthy child. The classic onset is fever-induced, prolonged, and generalized or hemiclonic seizure. The alternating character of unilateral seizures helps to differentiate DS from focal epilepsy. At an early stage, electroencephalographic studies and MRI usually do not reveal pathology [5]. In the course of the disease, patients aged 1 to 5 years develop pharmacoresistant seizures of multiple types, including focal, atypical absences, myoclonic, and atonic. Over time, seizure duration becomes shorter, but their frequency increases.

Interictal EEG evolves to background slowing and focal or generalized epileptiform discharges. Neuroimaging in older children and adults may reveal generalized atrophy or focal abnormalities, including hippocampal sclerosis [6]. Along with drug-resistant epilepsy, intellectual disability, behavioral, language, and sleep disorders, as well as gait abnormalities, are the most common comorbidities observed in patients diagnosed with DS [7,8,9]. Even though adults experience less frequent seizures, with a dominant pattern of nocturnal episodes, their quality of life is severely compromised.

In approximately 80–90% of cases, the syndrome is caused by de novo mutation in the voltage-gated sodium channel gene SCN1A, which results in the haploinsufficiency of Na_v_1.1, the alpha-1 subunit of the sodium channel. However, mutations in other genes (GABRA1, STXBP1, SCN9A, SCN1B, GABRG2, HCN1, CHD2) have also been described [10,11]. On the contrary, not all SCN1A mutations lead to Dravet Syndrome, but several variants have been found in cases of familial febrile seizures (FS), genetic epilepsy with febrile seizures plus (GEFS+), epilepsy of infancy with migrating focal seizures (EIMFS), or familial hemiplegic migraine type 3 (FHM3) [12,13,14]. The genotype–phenotype model recently published by Brunklaus et al. allows prediction of the development of Dravet Syndrome versus GEFS+ in SCN1A patients [15]. Based on factors such as age at seizure onset and a newly developed *SCN1A* genetic score, the model assists early recruitment of patients to precision therapies and facilitates family counseling.

Arising opportunities for genetic testing might facilitate early diagnosis, contributing to better developmental outcomes [16,17]. Despite the increasing availability of genetic testing and, therefore, early diagnosis, DS belongs to one of the most difficult epilepsies to manage, with poor neurodevelopmental outcomes. DS causes significant social and financial impacts on caregivers and health services, and moreover, is associated with increased premature mortality risk caused by consequences of status epilepticus and sudden unexpected death in epilepsy (SUDEP) [18,19].

In a multinational survey in 2017, less than 10% of the patients with DS reported freedom from seizures in the previous three months [16]. Recent discoveries of new effective treatments resulted in the need for new therapeutic recommendations. In this article, we describe current therapeutic approaches, recent changes, and promising therapies under investigation, which bring novel opportunities for patients with DS to reduce seizure activity and improve their quality of life.

## 2. Current Treatment Strategies

### 2.1. Limitation of Provoking Factors

Prompt, adequate treatment and reduction of seizure-provoking factors are key to minimizing the risk of status epilepticus and providing proper long-term management. Fever and hyperthermia are notable triggers in children and adults with DS [5]; therefore, avoiding high ambient temperatures, reduction of contact with sick persons, early use of antipyretics, and physical cooling methods are strongly recommended. Many patients are prone to seizures during exhaustion, and overexcitement is reported to be a common trigger, therefore, avoiding situations such as crowded, noisy assemblies helps to reduce the frequency of seizures [20]. Photosensitivity and visual patterns should also be considered triggering factors [21]. Vaccines are also known to be a seizure-precipitating factor with or without vaccine-associated fever [22]. Due to the COVID-19 pandemic, several SARS-CoV-2 vaccines have been approved recently. Although some case reports in the literature reveal neurological complications after COVID-19 vaccines, two surveys conducted in the UK and worldwide suggest that SARS-CoV-2 vaccines are well-tolerated in the DS population. [23,24].

### 2.2. Contraindicated Medications

It is broadly acknowledged that sodium channel blockers (e.g., carbamazepine, oxcarbazepine, lamotrigine, and phenytoin) may aggravate seizures in patients with Dravet syndrome and worsen cognitive outcomes [25]. This effect was initially described by Guerrini et al. [26]. Other anti-seizure medications such as vigabatrin, rufinamide, and phenobarbital may also exacerbate seizures in some patients with DS [25,27]. However, it has been reported that some patients may benefit from these agents, particularly lamotrigine, with observed exacerbation when weaning [28]. Thus, careful consideration must be given before therapeutic approaches with patients already taking sodium-channel blockers.

### 2.3. Acute Treatment

Prompt and efficient treatment, in cases of prolonged seizures and status epilepticus, prevents secondary brain damage and minimizes the mortality rate in DS. Administration of benzodiazepine compounds in both inpatients and outpatients remains the first line of acute treatment. In hospital settings, the intravenous route is preferred; otherwise, depending on availability, benzodiazepines might be administered via an intranasal, buccal, or intramuscular route with comparable efficacy [29,30]. Another promising route of administering alprazolam via a Staccato breath-actuated device has emerged in recent years [31]. While the usage of second-line agents such as phenytoin and phenobarbital is debatable, intravenous ganaxolone might be beneficial [32].

### 2.4. Chronic Management

Therapeutic approaches in DS have undergone tremendous changes in the last few years and are currently heading to more personalized treatment. The drugs which were recently approved for the treatment of the syndrome include stiripentol, fenfluramine, and cannabidiol.

#### 2.4.1. Stiripentol (STP)

Stiripentol received orphan drug status in the European Union in 2001, and in 2007, the EMA granted conditional marketing authorization, followed by approval by Japan and Canada in 2012 and the United States in 2018.

The drug inhibits the synaptosomal uptake of GABA and acts as an allosteric modulator of the GABAa receptor [33,34]. Stiripentol inhibits CYP450 isoenzymes, which leads to an increase in the concentration of other anti-seizure medications, including clobazam [35,36].

As an add-on therapy to VPA and CLB, stiripentol was demonstrated to reduce seizure frequency in two randomized, placebo-controlled trials [37,38]. Responders, which counted for participants with >50% reduction in the frequency of clonic or tonic-clonic seizures during the second month of the double-blind period compared to baseline, were 71% on the stiripentol arm and 5% on placebo. The second study showed similar results (67% of responders on stiripentol vs. 9% on placebo). The most common adverse events (stiripentol vs. placebo) included somnolence (67% vs. 23%), decreased appetite (46% vs. 10%), decreased weight (27% vs. 6%), agitation (27% vs. 16%), and hypotonia (18% vs. 13%). Neutropenia has been reported in some cases and was reversible by dosage reduction [39]. Long-term efficacy and safety of stiripentol were later determined in several observational studies, including patients with a wide age range, including adults [40,41,42,43].

#### 2.4.2. Fenfluramine (FFA)

Fenfluramine (Fintepla) was approved by the FDA and EMA in 2020 for the treatment of seizures in Dravet Syndrome. The drug was first used in the 1970s as an anorectic agent for patients with obesity, but it was withdrawn from the market in 1997 due to pulmonary hypertension and cardiac valvulopathy cases when used in high dosages [44].

The mechanism of action of fenfluramine is not fully elucidated. It has a high affinity for 5HT2A and 5HT2C receptors modulating the serotonergic pathway, but an additional effect on the sigma 1 receptor is also described [45,46,47].

Two prospective, double-blind, placebo-controlled trials demonstrated the efficacy and safety of fenfluramine in treating Dravet Syndrome [48,49]. In the first study, FFA was compared at two dosages (0.7 mg/kg/day and 0.2 mg/kg/day) with a placebo; patients on STP were not included. At both dosages, the effect, defined as mean monthly convulsive seizure frequency reduction (MCSF), was significantly higher in comparison to the placebo group (70% and 41%, respectively, versus 7.5%). In the second study, patients using 0.4 mg/kg/day fenfluramine as an add-on therapy to stiripentol attained a 54% reduction in monthly convulsive seizure frequency versus 5% with placebo. The responders’ rate for profound reduction (75% and more in MCSF) were 50% and 23% for higher and lower doses of FFA without STP, respectively, and 35% in the population study using STP. The most common adverse effects reported were decreased appetite, weight loss, diarrhea, fatigue, and somnolence. A cardiac follow-up did not reveal valvular heart disease or pulmonary hypertension. Therapy with FFA was also linked with prolonged seizure-free time intervals and provided a significantly higher number of seizure-free days [50]. Subsequent open-label extension studies and data from an access program confirmed the efficacy and safety of FFA in DS patients [51,52].

#### 2.4.3. Cannabidiol (CBD)

Although the effect of CBD on seizure reduction is not fully understood and is not specific to DS, Epidiolex/Epidyolex (a pure plant-derived CBD) was approved in 2018 in the United States and in 2019 in Europe for use in DS therapy as a reliable and quality-standardized product. CBD has also been approved as an adjunctive therapy in Lennox–Gastaut Syndrome (LGS) and tuberous sclerosis complex. The efficacy of cannabidiol was at first described in an open-label, prospective study of childhood-onset epilepsy of different etiology. Among patients included in the study, those diagnosed with Dravet Syndrome achieved a higher response than other subgroups, with a reduction of 69.2% and 46.7%, respectively, for the frequency of tonic and tonic-clonic seizures [53].

Due to the positive effect of the study, two randomized placebo-controlled trials (GWPCARE1 and GWPCARE2) were conducted to measure the efficacy of Epidiolex titrated to 20 mg/kg/day in seizure frequency reduction specifically in Dravet Syndrome [54,55]. They documented a significantly higher reduction of seizure frequency in the CBD group in comparison to the placebo group (39% vs. 13% in the total treatment period and 41% vs. 16% in the maintenance period). In the GWPCARE2 trial, two dosages (10 mg/kg/day and 20 mg/kg/day) were compared with placebo. The reported reduction from baseline was 48.7% and 45.6%, respectively. In both studies, patients treated with clobazam achieved higher reduction, probably due to an increased concentration of both compounds [56]. The most common adverse effects were similar in both studies and included somnolence, loss of appetite, and diarrhea [52,53]. Clinical practice and open-label extension studies have recently published evidence of CBD’s good tolerance and effectiveness [57,58,59,60].

Although several RCTs have proven the efficacy of stiripentol, fenfluramine, and cannabidiol, no comparison studies have been conducted. Due to new drugs being registered, but a lack of comparison studies, there was a strong need for therapeutical guidelines in DS [61,62]. In 2022, a multinational consensus based on the Delphi method was developed [63]; according to the consensus, valproic acid remains the first-line agent. Stiripentol, fenfluramine, and clobazam in different combinations should be added to VPA. Cannabidiol, with a lower efficacy in DS, remains behind the drugs mentioned. Ketogenic diets tend to not only reduce seizures but also improve cognitive outcomes [64]. In the case of failure to achieve a significant reduction of seizures by using ASM, vagal nerve stimulation should be considered. The dosing and therapeutic characteristics of recently approved therapeutic agents are presented in Table 1.

## 3. Novel Therapies

### 3.1. Modulators of Serotonin Signaling

A new spectrum of drugs modulating serotonin signaling have shown a potential to reduce seizure burden significantly. Results of ongoing studies on clemizole, lorcaserin, and trazodone might shift the current landscape of DS treatment into the more frequent use of these medicines, following the successful pathway of fenfluramine.

#### 3.1.1. Clemizole (EPX-100)

Clemizole is a first-generation antihistamine receptor antagonist discovered in the 1950s. It was identified as a potential therapeutic agent for the treatment of DS using scn1Lab mutant zebrafish [65,66]. Further study by Griffin et al. confirmed antiepileptic activity using a zebrafish model and revealed clemizole affinity for HTR2A and/or HTR2B receptors [46]. The phase I trial confirmed EPX-100 safety and tolerance among three sequential groups of eight healthy adult subjects each. Currently, male and female participants 2 years and older with DS are being recruited for a global, multicenter placebo-controlled phase II study (NCT04462770, Argus Trial) of clemizole hydrochloride as an add-on therapy.

#### 3.1.2. Lorcaserin (EPX-200)

EXP-200 is another serotonin signaling pathway agent and was approved by FDA under the trade name Belviq as a weight-loss medication [67]. It was also a subject of research for nicotine dependence and treatment of opioid and cannabis use disorders. Reduction of seizure activity was reported in a zebrafish model of DS. The mechanism of action depends on the activation of the HTR2C receptor, but it is not well understood. The drug was tested off-label in five pediatric patients (mean age 11.8 years, range: 7–18 years) with DS and showed a reduction in the number of seizures in all participants without serious adverse events causing cessation of therapy [46]. Another study by Tolete et al., in patients with DS (20/35 participants) and other severe epilepsy syndromes, reduced the mean monthly frequency of motor seizures by 43% from baseline [68]. The most common side effects reported in the severe epilepsy syndromes group were loss of appetite, weight loss, and decreased attentiveness. The MOMENTUM 1 study is currently recruiting patients above the age of 2. Lorcaserin was withdrawn from the market after an FDA alert due to higher malignancy rates observed in the treatment arm of a different epilepsy-related study (CAMELLIA-TIMI); therefore, the drug’s safety needs further evaluation.

#### 3.1.3. Trazodone (EPX-300)

Trazodone is another compound that has been proven to be capable of suppressing seizures in a DS zebrafish model [66], acting as a 5-HT receptor agonist or antagonist, depending on concentration. There are no past or ongoing clinical studies investigating trazodone in patients with DS.

### 3.2. Soticlestat (TAK-935/OV935)

Soticlestat is a selective inhibitor of cholesterol 24-hydroxylase (CH24H), whose efficacy for the treatment of CDKL5 deficiency, DS, and LGS (Lennox–Gastaut Syndrome) is being under investigation. The role of inappropriate cholesterol metabolism is being studied in seizure-related central nervous system disorders [69,70]. Soticlestat was proven to reduce seizure burden, protect against hyperthermia-induced seizures, and completely prevent SUDEP in heterozygous deletion of Scn1a mice models [71]. The safety and tolerance of the compound were confirmed in two phase I clinical trials [72,73]. A recently completed randomized, double-blind, placebo-control multicenter ELEKTRA study covered 51 pediatric patients diagnosed with DS and 88 with LGS. In the DS group, the 20-week weight-adjusted treatment schedule showed a median placebo-adjusted seizure frequency reduction of 46% from baseline [74]. The most frequent adverse events included lethargy and constipation. Currently, participants aged 2–21 years with DS are being recruited for a phase III randomized, double-blind, placebo-controlled clinical trial—SKYLINE (NCT04940624).

### 3.3. Gene Therapy

Disease-modifying compounds are expected to revolutionize current methods of treatment. Antisense nucleotides (ASOs), by successful delivery and modulation of target gene expression used in DEE mouse models, have shown efficacy in reducing seizures and risk of SUDEP [75,76], but further cognitive and behavioral outcomes need to be determined. This fact, together with a positive experience in the modulation of target gene expression in SMA and Huntington’s disease, indicates a promising perspective for precise, causative treatment in DS.

Viral gene therapy is the next expected milestone. Various research groups are developing new technologies suitable for use in DS, most of them based on adeno-associated viral vectors [77,78,79]. Promising pre-clinical data and kindred approaches in several CNS diseases have stimulated investigators’ direction for clinical trials. After confirmation of the efficacy of viral and non-viral gene methods, they may be replicated in the management of other channelopathies and developmental and epileptic encephalopathies.

#### 3.3.1. STK-001 (Antisense Oligonucleotides)

STK-001 is a compound generated by targeted augmentation of nuclear gene output (TANGO) using antisense oligonucleotides (ASOs). The most prominent example of ASO use is the approved treatment of spinal muscular atrophy using nusinersen (Spinraza). Clinical trials of different phases for other neurological diseases, including Huntington’s disease, Batten’s disease, amyotrophic lateral sclerosis, and different channelopathies, are underway. The goal of the therapy is not based on the reinstatement of functional gene copy. The mechanism of STK-0001 action depends on an increase of productive mRNA levels resulting in optimal sodium channel Nav1.1 protein expression, which is pathologically reduced in patients with DS [80].

A single intracerebroventricular dose of STK-001 in a DS mouse model showed an increased level of Nav1.1 protein and reduced SUDEP incidence [76]. Later, the same effect was confirmed in rats and non-human primates [81]. STK-001 is provided by a single-dose intrathecal administration in ongoing clinical trials. A phase II study (NCT04442295, MONARCH) using single or multiple ascending doses is currently recruiting patients to assess safety, pharmacokinetics, and seizure frequency. The first published results revealed median seizure frequency reductions of 17–37% from baseline. The effect was observed in 70.6% of the patients. The NCT04740476 (SWALLOWTAIL) study aims not only to evaluate long-term safety and tolerance of repeated doses of STK-001, but also to measure neurodevelopment, behavior, executive functions, and gait outcome. Another study (ADMIRAL) is being conducted in the UK, in which STK-001 is being administered in multiple doses to assess seizure frequency change and its effect on clinical status and quality of life.

#### 3.3.2. ETX101

The recent success of gene therapies, voretigine neparvovec (Loxturna) for Leber congenital amaurosis and onasemnogene abeparvovec (Zolgensma) for spinal muscular atrophy (SMA), have encouraged investigation into other therapeutic solutions for central nervous system diseases, based on AAV-9 vectors. The most advanced development was achieved by encoded therapeutics with AAV9-REGABA-eRFSCN1A. ETX101, as a non-replicating, recombinant adeno-associated viral vector serotype 9 (rAAV9) comprising a GABAergic regulatory element, was designed to upregulate SCN1A expression within GABAergic inhibitory interneurons. A single intracerebroventricular injection in DS mice model resulted in increased SCN1A mRNA transcripts and Nav1.1 protein levels in the brain, leading to a reduction of spontaneous and thermal-induced seizure frequency and elongated survival [82]. The safety and distribution throughout crucial brain regions were then confirmed in non-primate humans.

The first clinical trial of ETX101, called ENDEAVOR (NCT05419492), aims to enroll 22 SCN1A-positive Dravet Syndrome participants aged 6 to 36 months. The drug will be administered by a single-dose intracerebroventricular injection. It consists of an open-label part 1 to evaluate two dose levels and a randomized, double-blinded part 2 with a sham procedure. The estimated study start date is December 2022.

The list of ongoing clinical trials of novel therapeutic agents in DS is presented in Table 2.

## 4. Conclusions

**Figure 1:**
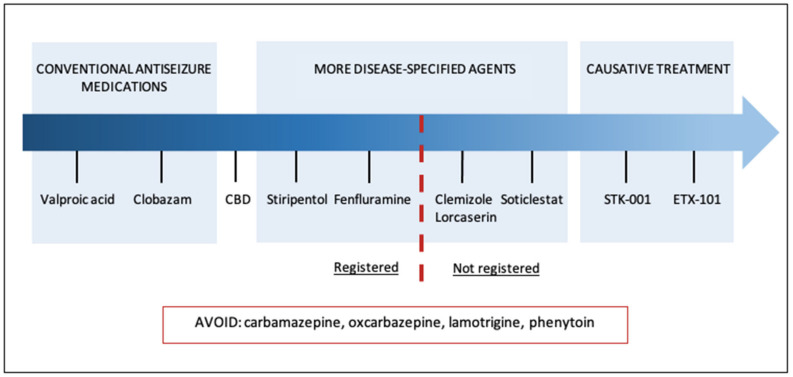
The landscape of Dravet Syndrome management.

Treatment approaches for Dravet Syndrome have evolved significantly in the last few years. Newly approved compounds have replaced less efficacious conventional drugs, and several promising trials are ongoing (Figure 1). However, a few limitations have hampered the improvement of DS care. Not all new therapies are approved and implemented in clinical practice. Accessibility and the limited number of comparable studies enhance the uncertainty about the most optimal therapy pattern. During concomitant use of several DS-approved drugs, caution about contraindications and drug-to-drug interactions is recommended. Moreover, novel therapies are mostly limited to clinical trials for relatively small cohorts of patients, with the possible exclusion of patients due to pre-existing humoral immunity to AAV.

Currently, individual preferences, patient characteristics, comorbidities, availability of the drugs armamentarium, and experts’ opinions remain the strongest determinants in therapy approach worldwide.

## Figures and Tables

**Table 1:** Dosing and therapeutic characteristics of new drugs approved in DS.

Drug	Dose			Adverse Events	Monitoring	Special Warnings
	Starting	Target	Max			
STP	10–15 mg/kg	Up to 50 mg/kg, average 25–30 mg/kg	3000 mg/d or 50 mg/kg	Decreased appetite and weight Insomnia Somnolence	CBC Liver function Weight	Neutropenia and thrombocytopenia
FFA	0.2 mg/kg	0.7 mg/kg/without STP 0.4 mg/kg/with STP	26 mg/d without STP 17 mg/d with STP	Decreased appetite Diarrhea Pyrexia	Echocardiography Weight	Valvulopathy
CBD	5 mg/kg	10 mg/kg	20 mg/kg	Somnolence Decreased appetite Diarrhea	Liver function	Liver dysfunction, Somnolence

Legend: STP—stiripentol, FFA—fenfluramine, CBD—cannabidiol.

**Table 2:** Ongoing clinical trials of novel therapeutic agents in DS.

Trial	Compound	Route, Dose	Mechanism of Action	Phase	Population Age
ENDEAVOR (NCT05419492)	ETX-001	Intracerebroventricular Dose-escalation design	Increase expression of endogenous SCN1A, specifically in GABAergic interneurons	I/II	6–36 months
NCT04442295, (MONARCH)	STK-001	Intrathecal injection	Increase production of SCN1A mRNA isoforms	II	2–18 years
NCT04740476 (SWALLOWTAIL)		Single ascending doses (10 mg, 20 mg, 30 mg, and 45 mg).			
NCT04442295 (ADMIRAL)		Multiple ascending doses—(20 mg, 30 mg, and 45 mg).			
NCT04462770 Argus Trial	EPX-100 (clemizole)	Oral	H1 antagonist with HTR2A and/or HTR2B receptors affinity	II	2 years and above
		Highest-tolerated dose titration			
NCT04572243 (MOMENTUM 1)	EPX-200 (lorcaserin)	Oral	HTR2C activator	III	2 years and above
		Up to 10, 20 mg/day for participants weighing 10 to <20, 20 to <40 kg, respectively.			
SKYLINE (NCT04940624).	TAK-935/OV935 (soticlestat)	Oral	selective inhibitor of cholesterol 24-hydroxylase	III	2–21 years
		Participants weighing <45 kg: soticlestat, mini-tablets, at the dose of 40 mg to 200 mg; participants weighing ≥45 kg: up to 300 mg			

## References

[1] Symonds J., Zuberi S.M., Stewart K., McLellan A., O’Regan M., MacLeod S., Jollands A., Joss S., Kirkpatrick M., Brunklaus A. (2019). Incidence and phenotypes of childhood-onset genetic epilepsies: A prospective population-based national cohort. Brain.

[2] Brunklaus A., Ellis R., Reavey E., Forbes G.H., Zuberi S.M. (2012). Prognostic, clinical and demographic features in SCN1A mutation-positive Dravet syndrome. Brain.

[3] Bayat A., Hjalgrim H., Møller R.S. (2015). The incidence of *SCN1A*-related Dravet syndrome in Denmark is 1:22,000: A population-based study from 2004 to 2009. Epilepsia.

[4] Wu Y.W., Sullivan J., McDaniel S.S., Meisler M.H., Walsh E.M., Li S.X., Kuzniewicz M.W. (2015). Incidence of Dravet Syndrome in a US Population. Pediatrics.

[5] Dravet C. (2011). The core Dravet syndrome phenotype. Epilepsia.

[6] Wirrell E.C., Laux L., Donner E., Jette N., Knupp K., Meskis M.A., Miller I., Sullivan J., Welborn M., Berg A.T. (2017). Optimizing the Diagnosis and Management of Dravet Syndrome: Recommendations from a North American Consensus Panel. Pediatr. Neurol..

[7] Catarino C.B., Liu J.Y., Liagkouras I., Gibbons V.S., Labrum R.W., Ellis R., Woodward C., Davis M.B., Smith S.J., Cross J.H. (2011). Dravet syndrome as epileptic encephalopathy: Evidence from long-term course and neuropathology. Brain.

[8] Takayama R., Fujiwara T., Shigematsu H., Imai K., Takahashi Y., Yamakawa K., Inoue Y. (2014). Long-term course of Dravet syndrome: A study from an epilepsy center in Japan. Epilepsia.

[9] Ouss L., Leunen D., Laschet J., Chemaly N., Barcia G., Losito E.M., Aouidad A., Barrault Z., Desguerre I., Breuillard D. (2018). Autism spectrum disorder and cognitive profile in children with Dravet syndrome: Delineation of a specific phenotype. Epilepsia Open.

[10] Cardenal-Munoz E., Auvin S., Villanueva V., Cross H.J., Zuberi S., Lagae L., Aibar J.A. (2022). Guidance on Dravet syndrome from infant to adult care: Roadmap for treatemnet planning in Europe. Epilepsia Open.

[11] Steel D., Symonds J.D., Zuberi S.M., Brunklaus A. (2017). Dravet syndrome and its mimics: Beyond *SCN1A*. Epilepsia.

[12] Mantegazza M., Gambardella A., Rusconi R., Schiavon E., Annesi F., Cassulini R.R., Labate A., Carrideo S., Chifari R., Canevini M.P. (2005). Identification of an Na _v_ 1.1 sodium channel (SCN1A) loss-of-function mutation associated with familial simple febrile seizures. Proc. Natl. Acad. Sci. USA.

[13] Escayg A., Macdonald B.T., Meisler M.H., Baulac S., Huberfeld G., An-Gourfinkel I., Brice A., LeGuern E., Moulard B., Chaigne D. (2000). Mutations of SCN1A, encoding a neuronal sodium channel, in two families with GEFS+2. Nat. Genet..

[14] Freilich E.R., Jones J.M., Gaillard W.D., Conry J.A., Tsuchida T.N., Reyes C., Dib-Hajj S., Waxman S.G., Meisler M.H., Pearl P.L. (2011). Novel SCN1A Mutation in a Proband w Malignant Migrating Partial Seizures of Infancy. Arch. Neurol..

[15] Brunklaus A., Pérez-Palma E., Ghanty I., Xinge J., Brilstra E., Ceulemans B., Chemaly N., de Lange I., Depienne C., Guerrini R. (2022). Development and Validation of a Prediction Model for Early Diagnosis of *SCN1A*-Related Epilepsies. Neurology.

[16] Lagae L., Brambilla I., Mingorance A., Gibson E., Battersby A. (2018). Quality of life and comorbidities associated with Dravet syndrome severity: A multinational cohort survey. Dev. Med. Child Neurol..

[17] O’Reilly H., Eltze C., Bennett K., Verhaert K., Webb R., Merrett A., Scott R.C., Whitney A., Cross J.H., de Haan M. (2018). Cognitive outcomes following epilepsy in infancy: A longitudinal community-based study. Epilepsia.

[18] Shmuely S., Sisodiya S.M., Gunning W.B., Sander J.W., Thijs R.D. (2016). Mortality in Dravet syndrome: A review. Epilepsy Behav..

[19] Gataullina S., Dulac O. (2017). From genotype to phenotype in Dravet disease. Seizure.

[20] Ceulemans B. (2011). Overall management of patients with Dravet syndrome. Dev. Med. Child Neurol..

[21] Genton P., Velizarova R., Dravet C. (2011). Dravet syndrome: The long-term outcome. Epilepsia.

[22] Tro-Baumann B., von Spiczak S., Lotte J., Bast T., Haberlandt E., Sassen R., Freund A., Leiz S., Stephani U., Boor R. (2011). A retrospective study of the relation between vaccination and occurrence of seizures in Dravet syndrome. Epilepsia.

[23] Clayton L.M., Balestrini S., Cross J.H., Wilson G., Eldred C., Evans H., Koepp M.J., Sisodiya S.M. (2021). The impact of SARS-CoV-2 vaccination in Dravet syndrome: A UK survey. Epilepsy Behav..

[24] Hood V., Berg A.T., Knupp K.G., Koh S., Laux L., Meskis M.A., Zulfiqar-Ali Q., Perry M.S., Scheffer I.E., Sullivan J. (2022). COVID-19 vaccine in patients with Dravet syndrome: Observations and real-world experiences. Epilepsia.

[25] De Lange I.M., Gunning B., Sonsma A.C.M., van Gemert L., van Kempen M., Verbeek N.E., Nicolai J., Knoers N.V.A.M., Koeleman B.P.C., Brilstra E.H. (2018). Influence of contraindicated medication use on cognitive outcome in Dravet syndrome and age at first afebrile seizure as a clinical predictor in *SCN1A* -related seizure phenotypes. Epilepsia.

[26] Guerrini R., Dravet C., Genton P., Belmonte A., Kaminska A., Dulac O. (1998). Lamotrigine and Seizure Aggravation in Severe Myoclonic Epilepsy. Epilepsia.

[27] Mueller A., Boor R., Coppola G., Striano P., Dahlin M., von Stuelpnagel C., Lotte J., Staudt M., Kluger G. (2011). Low long-term efficacy and tolerability of add-on rufinamide in patients with Dravet syndrome. Epilepsy Behav..

[28] Snoeijen-Schouwenaars F., Veendrick M., van Mierlo P., van Erp G., de Louw A., Kleine B., Schelhaas H., Tan I. (2015). Carbamazepine and oxcarbazepine in adult patients with Dravet syndrome: Friend or foe?. Seizure.

[29] Fişgin T., Gurer Y., Tezic T., Senbil N., Zorlu P., Okuyaz C., Akgün D. (2002). Effects of Intranasal Midazolam and Rectal Diazepam on Acute Convulsions in Children: Prospective Randomized Study. J. Child Neurol..

[30] Bhattacharyya M., Kalra V., Gulati S. (2006). Intranasal Midazolam vs Rectal Diazepam in Acute Childhood Seizures. Pediatr. Neurol..

[31] French J.A., Wechsler R., Gelfand M.A., Pollard J.R., Vazquez B., Friedman D., Gong L.H., Kamemoto E., Isojarvi J., Cassella J.V. (2019). Inhaled alprazolam rapidly suppresses epileptic activity in photosensitive participants. Epilepsia.

[32] Vaitkevicius H., Ramsay R.E., Swisher C.B., Husain A.M., Aimetti A., Gasior M. (2022). Intravenous ganaxolone for the treatment of refractory status epilepticus: Results from an open-label, dose-finding, phase 2 trial. Epilepsia.

[33] Poisson M., Huguet F., Savattier A., Bakri-Logeais F., Narcisse G. (1984). A new type of anticonvulsant, stiripentol. Pharmacological profile and neurochemical study. Arzneimittelforschung.

[34] Quilichini P.P., Chiron C., Ben-Ari Y., Gozlan H. (2006). Stiripentol, a Putative Antiepileptic Drug, Enhances the Duration of Opening of GABAA-Receptor Channels. Epilepsia.

[35] Tran A., Rey E., Pons G., Rousseau M., D’Athis P., Olive G., Mather G.G., Bishop F.E., Wurden C.J., Labroo R. (1997). Influence of stiripentol on cytochrome P450-mediated metabolic pathways in humans: In vitro and in vivo comparison and calculation of in vivo inhibition constants. Clin. Pharmacol. Ther..

[36] Giraud C., Treluyer J.-M., Rey E., Chiron C., Vincent J., Pons G., Tran A. (2006). In vitro and in vivo inhibitory effect of stiripentol on clobazam metabolism. Drug Metab. Dispos..

[37] Chiron C. (2019). Stiripentol for the treatment of seizures associated with Dravet syndrome. Expert Rev. Neurother..

[38] Chiron C., Marchand M.C., Tran A., Rey E., D’Athis P., Vincent J., Dulac O., Pons G., STICLO Study Group (2000). Stiripentol in severe myoclonic epilepsy in infancy: A randomised placebo-controlled syndrome-dedicated trial. Lancet.

[39] Chiron C., Helias M., Kaminska A., Laroche C., de Toffol B., Dulac O., Nabbout R., An I. (2018). Do children with Dravet syndrome continue to benefit from stiripentol for long through adulthood?. Epilepsia.

[40] Inoue Y., Ohtsuka Y. (2015). Long-term safety and efficacy of stiripentol for the treatment of Dravet syndrome: A multicenter, open-label study in Japan. Epilepsy Res..

[41] Yamada M., Suzuki K., Matsui D., Inoue Y., Ohtsuka Y. (2020). Long-term safety and effectiveness of stiripentol in patients with Dravet syndrome: Interim report of a post-marketing surveillance study in Japan. Epilepsy Res..

[42] Habermehl L., Mross P., Krause K., Immisch I., Chiru D., Zahnert F., Gorny I., Strzelczyk A., Rosenow F., Möller L. (2021). Stiripentol in the treatment of adults with focal epilepsy- a retrospective analysis. Seizure.

[43] Balestrini S., Sisodiya S.M. (2016). Audit of use of stiripentol in adults with Dravet syndrome. Acta Neurol. Scand..

[44] Abenhaim L., Moride Y., Brenot F., Rich S., Benichou J., Kurz X., Higenbottam T., Oakley C., Wouters E., Aubier M. (1996). Appetite-Suppressant Drugs and the Risk of Primary Pulmonary Hypertension. N. Engl. J. Med..

[45] Sourbron J., Smolders I., de Witte P., Lagae L. (2017). Pharmacological Analysis of the Anti-epileptic Mechanisms of Fenfluramine in scn1a Mutant Zebrafish. Front. Pharmacol..

[46] Griffin A., Hamling K.R., Knupp K., Hong S., Lee L.P., Baraban S.C. (2017). Clemizole and modulators of serotonin signalling suppress seizures in Dravet syndrome. Brain.

[47] Zhang Y., Kecskés A., Copmans D., Langlois M., Crawford A.D., Ceulemans B., Lagae L., de Witte P.A.M., Esguerra C.V. (2015). Pharmacological Characterization of an Antisense Knockdown Zebrafish Model of Dravet Syndrome: Inhibition of Epileptic Seizures by the Serotonin Agonist Fenfluramine. PLoS ONE.

[48] Lagae L., Sullivan J., Knupp K., Laux L., Polster T., Nikanorova M., Devinsky O., Cross J.H., Guerrini R., Talwar D. (2019). Fenfluramine hydrochloride for the treatment of seizures in Dravet syndrome: A randomised, double-blind, placebo-controlled trial. Lancet.

[49] Nabbout R., Mistry A., Zuberi S., Villeneuve N., Gil-Nagel A., Sanchez-Carpintero R., Stephani U., Laux L., Wirrell E., Knupp K. (2020). Fenfluramine for Treatment-Resistant Seizures in Patients With Dravet Syndrome Receiving Stiripentol-Inclusive Regimens. JAMA Neurol..

[50] Sullivan J., Specchio N., Devinsky O., Auvin S., Perry M.S., Strzelczyk A., Gil-Nagel A., Dai D., Galer B.S., Gammaitoni A.R. (2022). Fenfluramine significantly reduces day-to-day seizure burden by increasing number of seizure-free days and time between seizures in patients with Dravet syndrome: A time-to-event analysis. Epilepsia.

[51] Specchio N., Pietrafusa N., Doccini V., Trivisano M., Darra F., Ragona F., Cossu A., Spolverato S., Battaglia D., Quintiliani M. (2020). Efficacy and safety of Fenfluramine hydrochloride for the treatment of seizures in Dravet syndrome: A real-world study. Epilepsia.

[52] Strzelczyk A., Pringsheim M., Mayer T., Polster T., Klotz K.A., Muhle H., Alber M., Trollmann R., Spors H., Kluger G. (2021). Efficacy, tolerability, and retention of fenfluramine for the treatment of seizures in patients with Dravet syndrome: Compassionate use program in Germany. Epilepsia.

[53] Devinsky O., Marsh E., Friedman D., Thiele E., Laux L., Sullivan J., Miller I., Flamini R., Wilfong A., Filloux F. (2016). Cannabidiol in patients with treatment-resistant epilepsy: An open-label interventional trial. Lancet Neurol..

[54] Devinsky O., Cross J.H., Laux L., Marsh E., Miller I., Nabbout R., Scheffer I.E., Thiele E.A., Wright S. (2017). Trial of Cannabidiol for Drug-Resistant Seizures in the Dravet Syndrome. N. Engl. J. Med..

[55] Miller I., Scheffer I.E., Gunning B., Sanchez-Carpintero R., Gil-Nagel A., Perry M.S., Saneto R.P., Checketts D., Dunayevich E., Knappertz V. (2020). Dose-Ranging Effect of Adjunctive Oral Cannabidiol vs Placebo on Convulsive Seizure Frequency in Dravet Syndrome. JAMA Neurol..

[56] Geffrey A.L., Pollack S.F., Bruno P.L., Thiele E.A. (2015). Drug-drug interaction between clobazam and cannabidiol in children with refractory epilepsy. Epilepsia.

[57] Iannone L.F., Arena G., Battaglia D., Bisulli F., Bonanni P., Boni A., Canevini M.P., Cantalupo G., Cesaroni E., Contin M. (2021). Results from an Italian Expanded Access Program on Cannabidiol Treatment in Highly Refractory Dravet Syndrome and Lennox–Gastaut Syndrome. Front. Neurol..

[58] Laux L.C., Bebin E.M., Checketts D., Chez M., Flamini R., Marsh E.D., Miller I., Nichol K., Park Y., Segal E. (2019). Long-term safety and efficacy of cannabidiol in children and adults with treatment resistant Lennox-Gastaut syndrome or Dravet syndrome: Expanded access program results. Epilepsy Res..

[59] Scheffer I.E., Halford J.J., Miller I., Nabbout R., Sanchez-Carpintero R., Shiloh-Malawsky Y., Wong M., Zolnowska M., Checketts D., Dunayevich E. (2021). Add-on cannabidiol in patients with Dravet syndrome: Results of a long-term open-label extension trial. Epilepsia.

[60] Park Y.D., Linder D.F., Pope J., Flamini J.R., Moretz K., Diamond M.P., Long S.A. (2020). Long-term efficacy and safety of cannabidiol (CBD) in children with treatment-resistant epilepsy: Results from a state-based expanded access program. Epilepsy Behav..

[61] Cross J.H., Caraballo R.H., Nabbout R., Vigevano F., Guerrini R., Lagae L. (2019). Dravet syndrome: Treatment options and management of prolonged seizures. Epilepsia.

[62] Wheless J.W., Fulton S.P., Mudigoudar B.D. (2020). Dravet Syndrome: A Review of Current Management. Pediatr. Neurol..

[63] Wirrell E.C., Hood V., Knupp K.G., Meskis M.A., Nabbout R., Scheffer I.E., Wilmshurst J., Sullivan J. (2022). International consensus on diagnosis and management of Dravet syndrome. Epilepsia.

[64] Kossoff E.H., Zupec-Kania B.A., Auvin S., Ballaban-Gil K.R., Christina Bergqvist A.G., Blackford R., Buchhalter J.R., Caraballo R.H., Cross J.H., Dahlin M.G. (2018). Optimal clinical management of children receiving dietary therapies for epilepsy: Updated recommendations of the International Ketogenic Diet Study Group. Epilepsia Open.

[65] Baraban S.C. (2013). Forebrain electrophysiological recording in larval zebrafish. J Vis Exp..

[66] Dinday M.T., Baraban S.C. (2015). Large-Scale Phenotype-Based Antiepileptic Drug Screening in a Zebrafish Model of Dravet Syndrome. Eneuro.

[67] Higgins G.A., Fletcher P.J., Shanahan W.R. (2020). Lorcaserin: A review of its preclinical and clinical pharmacology and therapeutic potential. Pharmacol. Ther..

[68] Tolete P., Knupp K., Karlovich M., DeCarlo E., Bluvstein J., Conway E., Friedman D., Dugan P., Devinsky O. (2018). Lorcaserin therapy for severe epilepsy of childhood onset. Neurology.

[69] Nishi T., Kondo S., Miyamoto M., Watanabe S., Hasegawa S., Kondo S., Yano J., Watanabe E., Ishi T., Yoshikawa M. (2020). Soticlestat, a novel cholesterol 24-hydroxylase inhibitor shows a therapeutic potential for neural hyperexcitation in mice. Sci. Rep..

[70] Koike T., Constantinescu C.C., Ikeda S., Nishi T., Sunahara E., Miyamoto M., Cole P., Barret O., Alagille D., Papin C. (2022). Preclinical characterization of [18F]T-008, a novel PET imaging radioligand for cholesterol 24-hydroxylase. Eur. J. Nucl. Med..

[71] Hawkins N.A., Jurado M., Thaxton T.T., Duarte S.E., Barse L., Tatsukawa T., Yamakawa K., Nishi T., Kondo S., Miyamoto M. (2021). Soticlestat, a novel cholesterol 24-hydroxylase inhibitor, reduces seizures and premature death in Dravet syndrome mice. Epilepsia.

[72] Wang S., Chen G., Pich E.M., Affinito J., Cwik M., Faessel H. (2021). Safety, tolerability, pharmacokinetics, pharmacodynamics, bioavailability and food effect of single doses of soticlestat in healthy subjects. Br. J. Clin. Pharmacol..

[73] Wang S., Chen G., Pich E.M., Affinito J., Cwik M., Faessel H.M. (2022). Pharmacokinetics, pharmacodynamics and safety assessment of multiple doses of soticlestat in healthy volunteers. Br. J. Clin. Pharmacol..

[74] Hahn C.D., Jiang Y., Villanueva V., Zolnowska M., Arkilo D., Hsiao S., Asgharnejad M., Dlugos D. (2022). A phase 2, randomized, double-blind, placebo-controlled study to evaluate the efficacy and safety of soticlestat as adjunctive therapy in pediatric patients with Dravet syndrome or Lennox–Gastaut syndrome (ELEKTRA). Epilepsia.

[75] Lenk G.M., Jafar-Nejad P., Hill S.F., Huffman L.D., Smolen C.E., Wagnon J.L., Petit H., Yu W., Ziobro J., Bhatia K. (2020). *Scn8a* Antisense Oligonucleotide Is Protective in Mouse Models of *SCN8A* Encephalopathy and Dravet Syndrome. Ann. Neurol..

[76] Han Z., Chen C., Christiansen A., Ji S., Lin Q., Anumonwo C., Liu C., Leiser S.C., Aznarez I. (2020). Antisense oligonucleotides increase Scn1a expression and reduce seizures and SUDEP incidence in a mouse model of Dravet syndrome. Sci. Transl. Med..

[77] Mora-Jimenez L., Valencia M., Sanchez-Carpintero R., Tønnesen J., Fadila S., Rubinstein M., Gonzalez-Aparicio M., Bunuales M., Fernandez-Pierola E., Nicolas M.J. (2021). Transfer of SCN1A to the brain of adolescent mouse model of Dravet syndrome improves epileptic, motor, and behavioral manifestations. Mol. Ther. Nucleic Acids.

[78] Niibori Y., Lee S.J., Minassian B.A., Hampson D.R. (2020). Sexually Divergent Mortality and Partial Phenotypic Rescue After Gene Therapy in a Mouse Model of Dravet Syndrome. Hum. Gene Ther..

[79] Colasante G., Lignani G., Brusco S., Di Berardino C., Carpenter J., Giannelli S.G., Valassina N., Bido S., Ricci R., Castoldi V. (2020). dCas9-Based Scn1a Gene Activation Restores Inhibitory Interneuron Excitability and Attenuates Seizures in Dravet Syndrome Mice. Mol. Ther..

[80] Lim K.H., Han Z., Jeon H.Y., Kach J., Jing E., Weyn-Vanhentenryck S., Downs M., Corrionero A., Oh R., Scharner J. (2020). Antisense oligonucleotide modulation of non-productive alternative splicing upregulates gene expression. Nat. Commun..

[81] Meena M., Ticho B., Barriere O., Gosselin N. A pharmacokinetic (PK) model for STK-001, an antisense oligonucleotide (ASO), based on data from non-human primates (NHP) enables dose selection in patients with Dravet syndrome (DS) [Abstract 3.264]. Proceedings of the Annual Meeting of the American Epilepsy Society.

[82] Tanenhaus A., Stowe T., Young A., McLaughlin J., Aeran R., Lin I.W., Li J., Hosur R., Chen M., Leedy J. (2022). Cell-Selective Adeno-Associated Virus-Mediated *SCN1A* Gene Regulation Therapy Rescues Mortality and Seizure Phenotypes in a Dravet Syndrome Mouse Model and Is Well Tolerated in Nonhuman Primates. Hum. Gene Ther..

